# Microbial Inoculants for Improving Crop Quality and Human Health in Africa

**DOI:** 10.3389/fmicb.2018.02213

**Published:** 2018-09-19

**Authors:** Elizabeth Temitope Alori, Olubukola Oluranti Babalola

**Affiliations:** ^1^Department of Crop and Soil Science, Landmark University, Omu-Aran, Nigeria; ^2^Food Security and Safety Niche Area, Faculty of Natural and Agricultural Sciences, North-West University, Mahikeng, South Africa

**Keywords:** microbial inoculants, human health, biofertilizers, biocontrol agents, plant growth promoting rhizobacteria (PGPR)

## Abstract

Current agricultural practices depend heavily on chemical inputs (such as fertilizers, pesticides, herbicides, etc.) which, all things being equal cause a deleterious effect on the nutritional value of farm product and health of farm workers and consumers. Excessive and indiscriminate use of these chemicals have resulted in food contamination, weed and disease resistance and negative environmental outcomes which together have a significant impact on human health. Application of these chemical inputs promotes the accumulation of toxic compounds in soils. Chemical compounds are absorbed by most crops from soil. Several synthetic fertilizers contain acid radicals, such as hydrochloride and sulfuric radicals, and hence increase the soil acidity and adversely affect soil and plant health. Highly recalcitrant compounds can also be absorbed by some plants. Continuous consumption of such crops can lead to systematic disorders in humans. Quite a number of pesticides and herbicides have carcinogenicity potential. The increasing awareness of health challenges as a result of consumption of poor quality crops has led to a quest for new and improved technologies of improving both the quantity and quality of crop without jeopardizing human health. A reliable alternative to the use of chemical inputs is microbial inoculants that can act as biofertilizers, bioherbicide, biopesticides, and biocontrol agents. Microorganisms are able to carry out the plant growth promotion, pest and disease and weed control. Microbial inoculants are beneficiary microorganisms applied to either the soil or the plant in order to improve productivity and crop health. Microbial inoculants are natural-based products being widely used to control pests and improve the quality of the soil and crop, and hence human health. Microbial inoculants involve a blend of microorganisms that work with the soil and the soil life to improve soil fertility and health and by extension improve human health. Microbial inoculants have the ability to minimize the negative impact of chemical input and consequently increase the quantity and quality of farm produce. Microbial inoculants are environmental-friendly and deliver plant nutrients to plants in a more sustainable manner. Microbial inoculants can help reduce chemical fertilizer application. Microbial inoculants could include bacteria, fungi and algae. This research summarizes the impact of agricultural chemical inputs on human health. The contribution of microbial inoculants in sustainable maintenance of human health will be expatiated. Advances in microbial inoculants and technology and strategies to explore this natural, user friendly biological resource for sustainable maintenance of plant health will be discussed.

## Introduction

The advent of industrial system of agriculture involving the use of chemicals, preservatives, hormones, and antibiotics resulted in increased food growth and production (Alori and Fawole, 2017b). This new technique produces crop and livestock in larger quantities than the sustainable agriculture practiced in the past (Gilchrist et al., 2007). Industrial agriculture is characterized with mono cropping, in which the same crop is grown season after season. Mono cropping reduces the soil’s ability to naturally eliminate pests and replenish nutrients (Alori and Fawole, 2017a). To combat this menace industrial agriculture uses heavy doses of chemical fertilizers and pesticides (Alori et al., 2017a). Similarly, massive quantities of livestock such as cows, chickens, pigs, and turkeys are raised in confined, overcrowded and unsanitary conditions (Schmidt, 2002; Sayre, 2009).

Agrochemicals are commonly used in agricultural production to control or prevent diseases, pests and weeds in order to maintain high quality of agricultural products and eliminate or reduce yield losses. With this industrialized system, food is produced at reduced costs and farmers therefore get higher profits from their farm but serious concerns were being raised about health risks resulting from residues in drinking water and food and from occupational exposure (Alori and Fawole, 2017b). Suyal et al. (2016), reiterated that heavy doses of chemical fertilizer, although leading to self-reliance in food production, causes harmful impacts on living organisms and also depreciate the environment. The chemical contaminates the food produced and goes further to alter the normal body functions of the consumer (Sayre, 2009). Baker et al. (2002), reported 75% of pesticide residues in conventionally grown produce. Water supplies are polluted by toxic insecticides, herbicides, and chemical fertilizers used (Alori et al., 2017b).

One of the factors that increase susceptibility of farm workers to the injurious effects of agro-chemicals include language barrier. Most local farmers are illiterates; this hinders their understanding and adoption of safety precautions on labels and training in proper work practices. Lack of background in agriculture by most hired farm workers who use employment in the agricultural sector as an entry-level job may increase health and safety hazards in the agricultural workplace. It is therefore imperative to seek an alternative technology that will boost food production to meet the food requirements of the ever growing world population while minimizing the health hazards they pose to the environment, humans, and farm animals.

Microbial inoculants refer to formulations composed of beneficial microorganisms that play an important role in soil ecosystems for sustainable agriculture. Microbial inoculants are environmentally friendly and are a potential alternative to chemical fertilizers and pesticides (Babalola and Glick, 2012). They are composed of active strains of microorganisms which directly or indirectly stimulate microbial activity and hence improve mobility of nutrients from soil (Suyal et al., 2016). They could be phyto-stimulants, bio-fertilizers or microbial bio-control agents (Alori et al., 2017b). They provide protection against a range of different pathogens and they are effective bio-herbicides (Babalola, 2010b).

In view of these, this paper aims to summarize the impact of the conventional agricultural inputs (fertilizers, pesticides, and herbicides) on human health and the ameliorating effect of microbial inoculants on these hazards. Advances in microbial inoculants and technology and strategies to explore this natural, user friendly biological resource for sustainable maintenance of plant health will be discussed.

## Impact of Agricultural Inputs (Fertilizers, Pesticides and Herbicides) on Human Health

Due to increasing numbers of people in farming, in the vicinity of farming areas and consumers of contaminated farm products, agricultural chemical inputs have currently become major public health problems (Alori et al., 2017a,b). Agro-chemical runoff is a major contributor to surface-water contamination (Wohlfahrt et al., 2010). Excess and wrong usage of chemical fertilizers result in soil washing, contamination of ground water, streams, and sea (Önder et al., 2011). Agricultural chemical inputs gain access into human body systems through three major means: (1) oral ingestion, (ii) infiltration through the skin, and (iii) breathing (Roychowdhury et al., 2014). Pesticides have shown long-term resistance in food including vegetables, meat, and fruits, and in the human body (Battu et al., 2005). Quite a number of people are negatively affected by long-term exposure to agro-chemicals, even at low levels (Kirkhorn and Schenker, 2001). The illnesses range from respiratory disorders and musculoskeletal illnesses to dermal and cardiac related diseases. These illnesses are encountered by farm owners, operators, family members, and employees (Magauzi et al., 2011).

Factors that were responsible for the susceptibility of farm owners and workers to agrochemical poisoning include inadequate protective clothing and safety systems, lack of knowledge of the caution code for hazardous agrochemicals (Magauzi et al., 2011). The study in Nepal, India by (Bhandari, 2014), also confirmed that only 2.33% of the farm owners and workers had received training on the hazardous effect of agrochemicals and preventive measures to protect themselves. More also, in developing countries where less than 20% of the world agrochemical production are consumed, agrochemicals have been reported to account for 70% of acute poisoning among working population (United States Environmental Protection Agency [USEPA], 2016). In Nigeria, Ojo (2016), identified the following as factors that aggravate health hazards from pesticide use; the use of the cheaper but deadliest types of pesticides (in terms of persistence and toxicity); poor pesticide education leading to extensive misuse; pesticides residues on locally consumed products, poor legislation and lack of enforcement of available legislation; inadequate information, awareness and knowledge of the inherent dangers of pesticides and inadequacies in medical recognition and responses to pesticide poisoning and failure of regulatory systems.

Breast and prostate cancer have been linked to consumption of beef raised under industrial dairy systems where artificial growth hormones such as recombinant bovine growth hormone (rBGH) are administered to animals (Food and Water Watch, 2008). In intensive agriculture, antibiotics are administered to farm animals at unnatural levels and this has been reported to be responsible for some food related infections in human (Sayre, 2009).

**Table 1** provides a list of some health implication of some chemicals use in agriculture.

**Table 1:** Health implication of some chemicals use in agriculture.

S/N	Ailment	Type of agrochemical	Author
1	Cancer or carcinogenicity, birth defects, reproductive effects, liver or kidney damage, neurotoxicity, disruption of the endocrine system	Pesticides	Sharma and Singhvi, 2017
2	Heart attack, Cancer, Strokes, Bowel cancer	Fertilizers, pesticides, herbicides, etc.	Thippeswamy, 2013
3	Farmers Hypersensitivity Pneumonitis FHP/Farmer’s Lung Disease	Pesticides	Schenker, 1998
4	Nervous and reproductive system disorder	Pesticides	Benbrook, 2009
5	Asthma	Pesticides	Salam et al., 2004
6	Neurological deficits	Pesticides	Meggs and Langely, 1997
7	Headache, skin irritation, fatigue and eye irritation	Pesticides	Alvanaja et al., 1996
8	Oral-facial clefts	Pesticides	Nurminen, 1995
9	Congenital birth defects	Pesticides/fungicides	Garry et al., 1995
10	Limb reduction defects associated with organ system anomalies	Pesticides	Lin et al., 1994
11	Vomiting, Skin burn, itching, nausea, tiredness, and headaches	Pesticides	Tijani, 2006
12	Abdominal pain, cancer, restlessness, reproductive disorder, headaches excessive salivation, developmental disorder, and convulsions irritation of eyes	Pesticides	Adekunle et al., 2017

## Legislature and Pesticides Use in Nigeria

Nigerian farmers depend largely on agrochemicals such as fertilizers and pesticides for the control of many insect pests, weeds, and diseases. It was reported that about 125,000–130,000 metric tons of pesticides are applied annually in Nigeria (Asogwa and Dongo, 2009). Although organizations such as; National Agency for Foods and Drugs Control (NAFDAC), National Environmental Standards and Regulations Enforcement Agency (NESREA), Cocoa Research Institute of Nigeria (CRIN), and Nigeria Stored Products Research Institute (NSPRI) was established to ensure safe use of pesticides in Nigeria, implementation and enforcement of available policies and legislature are inadequate (Isaa, 2016).

Furthermore the Knowledge of Nigerian farmers on the risk associated with the use of pesticide is very low (Ojo, 2016), as a result, farmers would rather continue to use their time tested product that is deadly than complying with the law that places a ban on the use of such pesticide (Adegbola et al., 2011). Farmers do not take necessary precautions required to prevent dangers associated with the use of agrochemicals (Tijani, 2006). More also in Nigeria, pesticide residues in locally consumed products are not being monitored (Ojo, 2016).

The commonly used pesticide by Cocoa farmers in Ondo State of Nigeria as stated by Tijani (2006) include, Aldrex 20, Basudin, Peronox, Gammalin 20, Unden, Sandoz, Cacaobre Copper sulphate, and Thionex. Most of these chemicals were classified by World Health Organization (WHO) as highly or moderately hazardous Tijani, 2006).

## Contribution of Microbial Inoculant in Sustainable Maintenance of Human Health Viz a Vis

### Microbial Inoculants as Biofertilizers for Plant Growth, Yields and Nutritional Quality Enhancement

The need to increase agricultural production to meet the food requirement of the ever increasing world population makes consistent maintenance of soil fertility essential. Biofertilizers which are made up of active microbes are a viable alternative technology to increase food production without jeopardizing human and environmental health (Alori et al., 2017a). Biofertilizers include all organisms which supply or make different nutrients available to plants. Examples are nitrogen fixers, phosphorus solubilizers, potassium solubilizers, sulfur solubilizers, and mycorrhiza, etc. (Pathak and Kumar, 2016).

Substantial contribution of Biofertilizer in sustainable maintenance of human health has been reported. Biofertilizers improve the nutritious properties of fresh vegetables by increasing; the antioxidant activity, the total phenolic compounds and chlorophyll (Khalid et al., 2017). Spinach inoculated with different biofertilizers was found to have 58.72 and 51.43% higher total phenolic content than the un-inoculated control (Khalid et al., 2017). These secondary metabolites play preventive roles in cancer, neurodegenerative, and cardiovascular disorders (Rodríguez-Morató et al., 2015).

Inoculation of lettuce with *Azotobacter chroococcum* and *Glomus fasciculatum* also increased the total phenolic compounds, anthocyanins and carotenoids content of the vegetable (Baslam et al., 2011). Higher (48.02 and 40.46%) flavonoid content (antioxidant) was observed in lettuce co-inoculated with *G. fasciculatum* and *Glomus mosseae* (Baslam et al., 2011). Antioxidant biosynthesis by Arbuscular Mycorrhizal Fungi (AMF) has been reported (Carlsen et al., 2008; Nisha and RajeshKumar, 2010; Eftekhari et al., 2012). Taie et al. (2008), documented a 75% increase of phenolic acid biosynthesis in soybean seedlings inoculated with *Rhizobacteria.* Karthikeyan et al. (2010), found that inoculating *Pseudomonas fluorescens* and *Bacillus megaterium* into *Catharanthus roseus* significantly increased the alkaloid content of the crop.

Some researchers such as (Lakshmipathy et al., 2002; Selvaraj et al., 2008) also reported production of secondary metabolites like phenols, tannins, ortho-dihydroxy, flavonoids, phenols and alkaloids after inoculation of *G. mosseae*, *Bacillus coagulans* and *Trichoderma viride* to *Begonia malabarica* and *Calamus thwaitesii*. Health promoting compounds produced by biofertilizers according to other researchers include ferulic acid, flavonols (quercetin, kaempferol), caffeic acid, flavones (luteolin), coumaric acid isorhamnetin-3-gentiobioside-7-glucoside, and chlorogenic acid (Alarcón-Flores et al., 2014).

#### Mechanisms of Actions by Biofertilizers (PGPR)

Rhizosphere consists of bacteria named rhizobacteria which either directly or indirectly exert positive effects on plants (Kundan et al., 2015). These soil bacteria colonize plant root and benefit the plants by stimulating its growth and are therefore called plant growth promoting rhizobacteria (PGPR) (Beneduzi et al., 2012). PGPR stimulate plant growth and increase crop yields. PGPR are potential biofertilizers. They are less harmful to the environment and they also reduce the cost of chemical fertilizers. PGPR can also be referred to as plant health promoting rhizobacteria (PHPR) (Kundan et al., 2015). PGPR stimulate plant growth in two ways, directly and indirectly. The direct mechanism of plant growth by PGPR involves either providing the plant with a compound that is produced by the bacterium, such as phytohormones, or facilitating the uptake of certain nutrients from the environment (Glick, 1995b). Indirect mechanisms refer to the ability of PGPR to inhibit the deleterious effects of plant pathogenic organism through production of antagonistic substances or by inducing resistance to pathogens (Glick, 1995a).

#### Direct Mechanism

Direct mechanism includes phytohormones production, nitrogen fixation, increasing iron availability, phosphate solubilisation, siderophore, ammonia production, etc. PGPR carried out some of these functions via specific enzymes, which provoke morphological and physiological changes in plants thus enhance plant nutrient and water uptake.

##### Phytohormones production

Phytohormones are the chemical messengers that affect gene expression and transcription levels, cellular division and hence plant growth. Phytohormones affect seed germination, emergence of flowering, sex of flowers, senescence of leaves, and fruits (Kundan et al., 2015). Examples of phytohormones produced by PGPR include auxin. It is dominantly produced at the shoot apex, and are transported to the root apical meristem through the shoot vascular cambium and accrued in the quiescent center (QC), which is the columella initials and lateral root cap (Brunoud et al., 2012).

Indole-3-acetic acid (indole acetic acid, IAA) is one of the commonly studied auxins (Spaepen et al., 2007). Most times, IAA is produced in bud and young leaves of plant by several independent biosynthetic pathways (Kundan et al., 2015). It plays ultimate role in growth stimulation by being involved in DNA synthesis. The main function of IAA is cell division, differentiation, cell elongation, and extension. IAA causes a rapid increase in cell wall extensibility in young stems (Kundan et al., 2015). IAA promotes growth of auxiliary bud and bud formation. It also helps in the apical dominance, and also stimulates adventitious and lateral root development and growth (Grobelak et al., 2015). IAA plays important role in leaf and flower abscission (Kundan et al., 2015). Some other compounds like indole-3-acetamide, indole-3-pyruvate, indole-3-acetaldehyde, and 4-chloroindole-3-acetic acid have been reported to have auxin activity (Grobelak et al., 2015; Olanrewaju et al., 2017).

The effect of auxin produce by PGPR is determined by the concentration of plant-synthesized auxin. Hence auxin produced by a PGPR will stimulate root development in cases where the concentration of auxin produced by plant is suboptimal, but will inhibit root development in cases where the concentration of the plant produced auxin is already optimal (Spaepen et al., 2007). IAA is synthesized by at least three biosynthetic pathways and each is named for a key intermediate within the pathway. These pathways include: the indole acetamide (IAM) pathway, the indole pyruvic acid (IPyA) pathway, and the indole acetaldoxime (IAOx)/indoleacetonitrile (IAN) pathway (Duca et al., 2015). Many PGPR can have one, two, or even three functional IAA biosynthesis pathway (Olanrewaju et al., 2017).

Gibberellin (GA) is another phytohormone that has been observed in rhizobacteria. GAs are tetracyclic diterpenoid carboxylic acids with either C20 or C19 carbon skeletons (Hedden and Thomas, 2012). Even though 136 globberelline structures have been identified, only four have been identified in bacteria (Hedden and Thomas, 2012). GAs activate important growth processes such seed germination, stem elongation, flowering, and fruit setting (Zaidi et al., 2015). They improve photosynthesis rate, and chlorophyll content (Khan et al., 2015). GAs stimulate shoot growth and inhibit root growth via the actions of the GA signaling system, and the DELLA repressor which trigger GA-inducing genes (Martínez et al., 2016).

##### 1-Aminocyclopropane-1-carboxylate (ACC) deaminase

1-Aminocyclopropane-1-carboxylate deaminase is another enzyme produced by some PGPR which facilitate plant growth and development by decreasing ethylene levels. Ethylene is a plant growth hormone produced by approximately all plants and also by different biotic and abiotic processes in soils. Ethylene induces multifarious physiological changes in plants (Ahemad and Kibret, 2014). Ethylene is a growth hormone that has also been established as a stress hormone (Gamalero and Glick, 2015). Biotic and abiotic stress such as insect and nematode damage, drought or flood, presence of metals, chemicals (both organic and inorganic), ultraviolet light, extreme temperatures, mechanical wounding as well as fungal and bacterial pathogens triggers increased production of ethylene in plants (Ali et al., 2014). However, its production beyond the threshold levels in plant tissue affects the shoot and root development in plant negatively, but ACC deaminase produced by PGPR will reduce ethylene levels by converting ACC (ethylene precursor) to α-Ketobutyrate and ammonia and thereby restoring normal plant development (Olanrewaju et al., 2017). Prior application of ACC deaminase-containing PGPR (microbial inoculant) to plants typically reduce the concentration of ethylene produced by the plants as a result of stress and thereby decreases the damage that the plant incurs from the stress (Glick, 2012).

##### Siderophores production

Siderophores are small peptidic molecules which contain side chains and functional groups that provide a high-affinity set of ligands to which ferric ions can bind (Goswami et al., 2016). Microorganisms evolved these highly specific pathways to satisfy nutritional requirements of iron (Beneduzi et al., 2012). Siderophores producing microbes can therefore be classified into four main classes (based on their iron-coordinating functional groups, structural features and type of ligands) namely; carboxylate, hydroxamates, phenol catecholates, and pyoverdines (Crowley, 2006). Bacteria siderophore can prevent or lessen proliferation of pathogen by reducing the amount of iron that is available to a pathogen (Shen et al., 2013).

Siderophore producing PGPR therefore has competitive advantages over other microorganisms in the rhizosphere (Haas and Défago, 2005). Siderophores produced by *Chryseobacterium* spp. C138 when delivered to the root were effective in the supply of iron in tomato plant (Radzki et al., 2013). Likewise, Siderophore producing *Pseudomonas* strain showed significant increase in germination and plant growth (Sharma and Johri, 2003).

##### Nitrogen fixation

Nitrogen is the most important plant nutrient required for growth and productivity. This is because nitrogen is the basic building block of plant, animal and microorganisms. Nitrogen fixation is the conversion of molecular or atmospheric nitrogen into form utilizable to plant by nitrogen fixing microorganisms using an enzyme system called nitrogenase (Kim and Rees, 1994). This is also known as Biological nitrogen fixation (BNF). BNF mostly occurs at mild temperatures (Raymond et al., 2004). This process consumes significant amount of energy in the form of ATP. The nitrogenase gene (nif) required for BNF is sensitive to oxygen; to therefore prevent oxygen from inhibiting nitrogen fixation while at the same time providing sufficient oxygen for the bacteroides within the nodule to respire, it is essential that bacterial hemoglobin which can bind free oxygen is introduced (Kundan et al., 2015). It has also been reported that the nif genes consist of structural genes which activate Fe protein, molybdenum, and other regulatory genes which are directly involved in the synthesis and functions of enzyme and they are present in both symbiotic and free living systems (Kundan et al., 2015).

Biological nitrogen fixation include both symbiotic nitrogen fixation and the free living nitrogen fixing system. Symbiotic nitrogen fixers include the following genera, *Rhizobium*, *Achromobacter*, *Sinorhizobium*, *Azoarcus*, *Mesorhizobium*, *Frankia*, *Allorhizobium*, *Bradyrhizobium*, *Burkholderia*, *Azorhizobium*, and *Herbaspirillum* (Babalola, 2010a; Pérez-Montaño et al., 2014; Turan et al., 2016). Some of the important non-symbiotic nitrogen-fixing bacteria include: *Azoarcus* sp., *Herbaspirillum* sp., *Gluconacetobacter diazotrophicus*, and *Azotobacter* sp. (Vessey, 2003).

##### Ammonia production

The soil consists of plant, microbial, and animal residues. The quantitatively most important N containing molecules in the residue are proteins; chitin and peptidoglycan with the proteins alone comprise 60% or more of the N in plant and microbial cells (Sinha, 2014). Organic nitrogen residues in soil organic matter is converted by some PGPR such as the ammonia nitrifyers like *Pseudomonas* sp. and *Bacillus* sp. to amino acid and the amino acid is then digested to produce ammonia through the process called ammonification (Geisselera et al., 2010). This is a very important biochemical process in soil because some soil bacteria use the ammonia produced to build their own body protein while some other soil bacteria convert the ammonia to nitrite, e.g., *Nitrobacter* sp. and then to nitrate, e.g., *Nitrosomonas* sp. Still other bacteria can reduce ammonia to Nitrogen gas (Alacamo, 2001).

##### Phosphorus solubilisation

Phosphorus (P) is an essential element that is necessary for plant growth and development and it is second only to nitrogen (Azziz et al., 2012). P occurs in soil in both organic and inorganic forms which are not available to plant. However, a number of PGPR have been reported to mobilize poorly available phosphorus via solubilisation and mineralization. Examples include *Pseudomonas* spp., *Agrobacterium* spp., *Bacillus circulans*, *Azotobacter* spp., *Bacillus* spp., *Burkholderia* spp., *Enterobacter* spp., *Erwinia* spp., *Kushneria* spp., *Paenibacillus* spp., *Ralstonia* spp., *Rhizobium* spp., *Rhodococcus* spp., *Serratia* spp., *Bradyrhizobium* spp., *Salmonella* spp., *Sinomonas* spp., and *Thiobacillus* spp. (Alori et al., 2017b). The mechanism employed by Phosphorus solubilizing bacteria in promoting plant growth include production of plant growth hormones, promoting the efficiency of BNF and enhancing the availability of some nutrient elements such as iron, zinc, etc. (Wani et al., 2007).

The most important mechanism of inorganic phosphorus solubilisation by PGPR is the production of mineral dissolving compounds such as organic acids, hydroxyl ions, protons, and CO_2_ (Sharma et al., 2013). Some other mechanisms of mineral phosphate solubilization by PGPR include the production of inorganic acids (such as sulphuric, nitric, and carbonic acids), the production of chelating substances and enzymolysis or liberation of enzymes (Zhu et al., 2011; Alori et al., 2017b).

#### Indirect Mechanisms

Indirect mechanism refers to the ability of PGPR to reduce the deleterious effects of plant pathogens on the growth of crop. This involves synthesizing antibiotics, induced systemic resistance (ISR), synthesizing hydrogen cyanide (HCN), competition and producing lytic enzymes including chitinases, proteases, cellulases, lipases, and 1,3-glucanases that can lyse a portion of the cell walls of many pathogenic fungi. These mechanisms are difficult to study in the system and they are therefore considered critical (Kundan et al., 2015).

##### Production of antibiotics

The major mechanism employed by PGPR to combat deleterious effects of plant pathogens is the production of one or more antibiotics (Raaijmakers and Mazzola, 2012). Antibiotics are low molecular weight compounds that are produced by PGPR which are deleterious and critical to important enzymes and metabolism of other microorganisms and thus retard the growth (Kundan et al., 2015). Some plant pathogens can develop resistance against specific antibiotics hence the ability of PGPR to produce one or more antibiotics, enhance their ability to act as effective antagonistic agents against plant pathogens (Glick et al., 2007). Antibiotics produced by antagonistic microbes have biostatic and biocidal effects on soil-borne plant pathogens (Bhattacharyya et al., 2016).

However, Olanrewaju et al. (2017), noted that an antibiotic that is observed to control a pathogen might not be as much effective against another pathogen on the same plant. Likewise, the antibiotic-producing PGPR may exhibit varying differences in its actions at different field conditions. Also, the activity of a biocontrol bacterium can be altered by the method of cultivation and formulation of the biocontrol PGPR in the laboratory and by its mode of application (Glick, 2015).

*Bacillus* and *Pseudomonas* spp. have been recognized to produce a variety of antibiotics such as tas A, subtilin, bacilysin, sublancin, iturin, chlorotetain, fengycin, subtilosin, and bacillaene (from *Bacillus* spp.). Phenazine-1-carboxylic acid (PCA), Zwittermycin A, Cepaciamide A, Karalicin, Pseudomonic acid, Kanosamine, Rhamnolipids, Cepafungins, Azomycin, Butyrolactones, 2,4-Diacetyl Phloroglucinol (DAPG), Aerugine, Pyrrolnitrin and Oomycin A (from *Pseudomonas* spp.) (Goswami et al., 2016).

##### Induced systemic resistance

Induced systemic resistance is a mechanism in which non-pathogenic microbes, such as PGPR, reduce the deleterious effects of plant pathogens by stimulating a resistance mechanism in the plants (Van Loon et al., 1998). This mechanism increases resistance at the particular sites of plants at which induction had occurred, i.e., the defense mechanism of ISR is activated only when there is an attack of pathogenic agent (Kundan et al., 2015). ISR is not pathogen specific but rather top the plant against a range of different pathogens. Jasmonate and ethylene are plant hormones that stimulate plants defense response to pathogens, hence ISR employ these hormones to stimulate resistance mechanism in the plants (Verhagen et al., 2004). It activates the “dormant” defense mechanisms which become expressed in response to external contacts from pathogens or insect. PGPR contribute to sustaining the intrinsic resistance of plant to pathogenic organisms (Enebe and Babalola, 2018).

Plant protection by ISR is controlled by a network of coordinated signaling pathways and these are dominated and regulated by plant hormones sharing signaling components (Pieterse et al., 2014). ISR is regulated by the redox-regulated protein non-expressor of PR genes1 (NPR1) which is produced in the cytoplasm as an oligomer via intermolecular disulfide bonds (Olanrewaju et al., 2017). Many PGPR has been documented to activate ethylene dependent ISR (Weller et al., 2012; Lucas et al., 2014).

##### HCN production

Hydrogen cyanide are secondary metabolite that acts as an effective agent for the biocontrol of weeds. HCN produced by PGPR has the ability to inhibit electron transport chain and energy supply to cell, resulting to death of cells. HCN producing rhizobacteria are therefore effective agent of biological weed control (Kundan et al., 2015). Biocontrol PGPR that produces HCN can also synthesize some cell wall degrading enzymes or antibiotics (Ramette et al., 2006). HCN can also act as an anti-fungi agent. HCN synthesized by PGPR is usually in small quantity, this ensures that the fungi do not develop resistance to the synthesized antifungal thereby enhancing the effectiveness of antifungal (Olanrewaju et al., 2017). HCN ability to inhibit important metalloenzymes including cytochrome c oxidase affects its toxicity effectiveness (Nandi et al., 2017).

##### Competition

**FIGURE 1:**
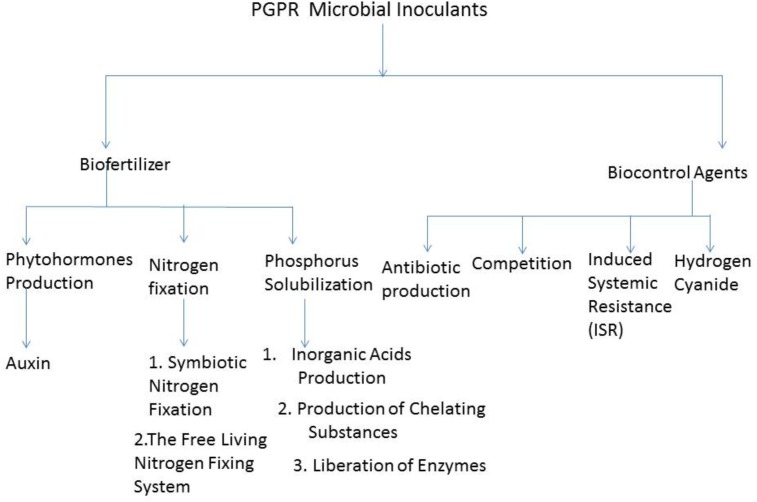
Schematic representation of some importance of Microbial Inoculant in Agriculture and the Mechanism of Actions.

Plant growth promoting rhizobacteria can limit the proliferation of pathogenic organisms by competing with them for the sparsely available nutrients. Barahona et al. (2011), reported that some biocontrol PGPR outcompete plant pathogens, either for binding sites on the plant root or for nutrient. As a result, limit the binding of the pathogen to the plant and thereby making it difficult for it to proliferate. However, it has been documented that PGPR competitiveness works in synergy with other biocontrol mechanisms to inhibit the functioning of phytopathogens (Olanrewaju et al., 2017). **Figure 1** shows the schematic representation of some of the importance of microbial inoculants in agriculture and their mechanism of actions.

### Microbial Inoculants as Biocontrol Agents (Biopesticides, Bioherbicides, Biofungicides)

Many microorganisms demonstrate antifungal and antibacterial activity and are therefore used as biopesticides (Rani et al., 2018). Microbial inoculants play a critical role in biocontrol technology employed in agricultural ecosystems. The mechanisms of biocontrol exercised by most microbial inoculants could be attributed to release of extracellular hydrolytic enzymes, competition for nutrients and secondary metabolites toxic to plant pathogens at very low concentrations (Rani et al., 2018), while some induce defense responses such as Systemic Acquired Resistance in the host plants (Mathivanan et al., 2008). These organisms help in reducing damage to the plants from pathogenic agents and can also modulate the levels of some plant hormones like ethylene and auxin (Glick, 1995a). Beneficial effects of microbial inoculants on plants include control of fungal infections (Babalola and Glick, 2012). Biological control activity exhibited by some microbial inoculants includes herbicidal activity; examples of which include *Colletotrichum coccodes* a mycoherbicide of velvet leaf, biofungicide of *Fusarium* spp. (Babalola, 2007) and mycoherbicides of Striga (Zahran et al., 2008). *Trichoderma harzianum* by producing volatile antibiotics inhibit wood rots and other fungal plant pathogens by up to 60% (Harman et al., 2004).

*Aspergillus fumigatus*, *Aspergillus niger*, *Penicillium funiculosum*, *Penicillium aurantiogriseum*, *Penicillium citrinum*, and *Trichoderma koningii* have been reported to be effective against plant pathogenic fungi *Phytophthora infestans* (Rani et al., 2007). More also, Cavaglieri et al. (2005), Pereira et al. (2007), Etcheverry et al. (2009), and (Nesci et al., 2005) have also reported *Bacillus amyloliquefaciens*, *Amphibacillus xylanus*, *Microbacterium oleovorans*, and *Sporolactobacillus inulinus* to show growth inhibition against fungi pathogens. *Bacillus subtilis* was reported to control *Aspergillus flavus* and aflatoxin production both in the field and in the store (Kimura and Hirano, 1988; Nesci et al., 2005). *Mitsuaria* sp. provide a biocontrol effect on bacterial leaf spot (Cepeda, 2012). Pseudomonads were also reported to exhibit biocontrol effect on *Fusarium* wilts (Cepeda, 2012). *Bacillus* spp. has the capacity to produce inhibitory volatile substances and have therefore been reported to be effective in the biological control of microbial diseases in a wide range of plants (Cepeda, 2012). Rhizobia group showed positive effects as biocontrol agents against *Pythium* disease (Antoun and Prévost, 2005).

### Microbial Inoculants in Food Processing

Microbial inoculants are employed in food processing to improve the nutritional value and food properties such as aroma, taste, texture, safety, and shelf-life (Vitorino and Bessa, 2017). Microbial inoculants are also used for food fermentation and preservation (Borneman et al., 2013). Quite a number of value adding products like vitamins, flavor compounds, enzymes, dyes, and food ingredients are produced by the application of microbial inoculants (Lipkie et al., 2016; Vitorino and Bessa, 2017). Some important pharmacological molecules are also produced using microbial inoculants (Vitorino and Bessa, 2017).

The use of microbial inoculants in food processes improves process efficiency by promoting process control, safety, product quality, yield, and consistency. Many microorganisms have been reported for use in the food and drug processing industries, examples include; *Aspergillus* spp. which is utilized in alcoholic beverage production. Citric acid produced by *A. niger* is used in food preservation (Mojsov, 2016). Many other microbes are capable of producing polyunsaturated fatty acids, flavoring agents used in food formulations, certain complex carbohydrates and amino acids like lysine and glutamic acid (FAO, 2010). More advantages such as lower production costs, the possibility of large-scale production in industrial fermenters, the possibility of genetic manipulation, and rapid culture development are obtained from enzymes sourced from microbes than those from vegetables and animals (Lipkie et al., 2016).

## Forms and Methods of Application of Microbial Inoculants in Food Production

Microbial inoculants could exist in various forms such as solid or liquid. It could be made up of bacteria or fungi. It could also consist of a pure culture or a mixed culture (Reddy and Saravanan, 2013). There can be various carriers such as peat, clay, and fly ash, coal, saw dust, wheat bran, peat supplemented with chitin-containing materials, inorganic materials such as vermiculite, perlite, silicates, kaolin, and betonies. Carriers for preparing inocula should be designed to provide a favorable microenvironment for the PGPM to ensure their viability and adequate shelf life of the inoculant formulation (preferably 2 months or more at room temperature). A desirable carrier should be easily available, stable, economical, eco-friendly, easy to apply, and have good moisture-holding capacity and pH-buffering capacity (Malusá et al., 2012).

Carrier-type also determines the form of the inoculant (solid or liquid). In the case of solid inocula, the size of the granules or beads used for immobilization of the microbe may vary from 75 to 250 μm (Malusá et al., 2012). Liquid inoculants can be broth cultures, suspensions in solutions of humic acid, or suspensions in mineral or organic oils or oil-in-water suspensions. Liquid or powder-type inoculants can be used to coat the seeds, for root dipping at the time of transplantation of seedlings, or can be applied directly into the furrow (or seed beds) or as a foliar spray (Reddy and Saravanan, 2013).

## Challenges to the Conventional Application of Microbial Inoculants in Agriculture

Despite the several advantages of microbial inoculant technology over the use of agro-chemicals, it’s wide spread utilization is limited by the following challenges. Microbial inoculants have been applied (mainly in research) in the forms of liquids (as sprays, root dips, drenches) or as dry formulations with huge successes recorded, but most of these techniques are not practicable on a large scale. This is because large amount are required for optimum functionality of the inoculants (Callaghan, 2016). PGPR are highly selective and targeted unlike chemical inputs that are broad spectrum product. It only impacts a selected or targeted organism. This therefore results in inconsistency of quality and efficacy under field conditions comprising various organisms act simultaneously (Timmusk et al., 2017).

Another success-limiting factor in the universal utilization of microbial inoculant in agriculture is the variability in shelf-life. It is a serious challenge maintaining viability of microbes present in microbial inoculant formulations (Callaghan, 2016). The viability of microbes in inoculated seeds varied significantly with treatment method and storage temperature. Extended survival of microbial inoculants at ambient storage conditions is recommended for microbial inoculant to become part of the mainstream agriculture. More-also, as reported by (Callaghan, 2016), the cost of maintaining the viability of both seeds and the microbes during storage is very high.

Furthermore, the use of some microbial inoculants as biocontrol agents can be highly risky. This is because some microbial biocontrol agents have been reported toxic and pathogenic to non-target organisms (Cuddeford and Kabaluk, 2010).

## Conclusion

Food production by the use of microbial inoculants is a viable alternative to destructive health effects caused by consumption of food produce by the use of agrochemicals such as pesticides, inorganic fertilizers, herbicides, etc. The knowledge of the mechanisms of actions employed by microbial inoculants will play a vital role in their use in sustainable agriculture. Use of chemicals in agriculture can be avoided and thus they can be removed from human diets. Pest and weed control can be achieved by employing microbial inoculants as bio-control agents and bio-herbicides. Harnessing natural resources including beneficiary microorganisms is one of the most effective approaches to improving farm productivity and food quality in a sustainable way. Microbial inoculant technology will ensure healthy food security for the future population. We also suggest that those at the helm of authority should review pesticide laws to enhance the effective supervision of pesticide quality and monitoring of existing laws on the use of agrochemicals. There is need to also educate the farmers on the danger associated with indiscriminate use of agrochemicals.

## Author Contributions

All authors contributed equally and made a substantial, direct and intellectual contribution to the work, and approved it for publication.

## Conflict of Interest Statement

The authors declare that the research was conducted in the absence of any commercial or financial relationships that could be construed as a potential conflict of interest. The handling Editor declared a past co-authorship with one of the authors OB.
